# Hypothesis-based food, feed, and environmental safety assessment of GM crops: A case study using maize event DP-202216-6

**DOI:** 10.1080/21645698.2020.1869492

**Published:** 2021-01-21

**Authors:** Jennifer A Anderson, Rod A. Herman, Anne Carlson, Carey Mathesius, Carl Maxwell, Henry Mirsky, Jason Roper, Brenda Smith, Carl Walker, Jingrui Wu

**Affiliations:** Corteva Agriscience™, Johnston, Iowa, USA

**Keywords:** Human safety, environmental safety, yield potential, ZMM28, genetically modified, safety assessment, *Zea mays* L

## Abstract

Event DP-2Ø2216-6 (referred to as DP202216 maize) was genetically modified to increase and extend the expression of the introduced *zmm28* gene relative to endogenous *zmm28* gene expression, resulting in plants with enhanced grain yield potential. The *zmm28* gene expresses the ZMM28 protein, a MADS-box transcription factor. The safety assessment of DP202216 maize included an assessment of the potential hazard of the ZMM28 protein, as well as an assessment of potential unintended effects of the genetic insertion on agronomics, composition, and nutrition. The history of safe use (HOSU) of the ZMM28 protein was evaluated and a bioinformatics approach was used to compare the deduced amino acid sequence of the ZMM28 protein to databases of known allergens and toxins. Based on HOSU and the bioinformatics assessment, the ZMM28 protein was determined to be unlikely to be either allergenic or toxic to humans. The composition of DP202216 maize forage and grain was comparable to non-modified forage and grain, with no unintended effects on nutrition or food and feed safety. Additionally, feeding studies with broiler chickens and rats demonstrated a low likelihood of unintentional alterations in nutrition and low potential for adverse effects. Furthermore, the agronomics observed for DP202216 maize and non-modified maize were comparable, indicating that the likelihood of increased weediness or invasiveness of DP202216 maize in the environment is low. This comprehensive review serves as a reference for regulatory agencies and decision-makers in countries where authorization of DP202216 maize will be pursued, and for others interested in food, feed, and environmental safety.

## Introduction

Safety assessments are conducted to characterize the food, feed, and environmental safety of genetically modified (GM) crops prior to commercial approval.^1–3^ To assess the food and feed safety of a newly introduced protein in a GM plant, a tiered weight of evidence approach is used, which first identifies potential hazards (Tier I) by considering the source and history of safe use (HOSU) of the gene, the mode of action of the protein, and potential for the protein to be an allergen or toxin (via bioinformatics assessment of the gene sequence compared with a database of known allergens or toxins, and an assessment of protein stability to heat and digestion). When triggered by uncertainty in the Tier I assessment, additional hazard characterization studies (for example, an acute toxicity study; Tier II) may be warranted.^4^ In many cases, not all Tier I and Tier II data are scientifically justified to assess the safety of a newly expressed protein. As originally proposed in Ref. 4, these studies should only be required on a case-by-case basis when these data are informative to the overall risk assessment.

A science-based, WOE approach was used to assess the food, feed, and environmental safety of DP-2Ø2216-6 maize (referred to as DP202216). This review of the safety of DP202216 maize serves as a reference for regulatory agencies and decision-makers in countries where authorization of DP202216 maize will be pursued, and for others interested in food, feed, and environmental safety. Furthermore, this case study demonstrates the suitability of using this science-based streamlined approach for hypothesis-driven regulatory oversight of GM crops.

## Characteristics of the Introduced Trait

Since the early 1930s, maize grain yields have been incrementally increasing, largely as a result of the widespread adoption of hybrid crops, germplasm improvement through selective breeding, and optimized crop management practices.^5^ Over thousands of breeding cycles, desired plant phenotypes and their underlying genotypes have been selected by breeders, and the expression of endogenous maize genes has been shaped by these decisions over time.^6^ In addition to naturally occurring mutations, native genes have been modified using radiation and chemical mutagenesis,^7,8^ which adds genetic variability for selection. Recently, biotechnology tools have been adopted to help identify and target specific maize genes that are associated with yield or yield-related phenotypes [for example, the *zmm28* gene, which encodes the ZMM28 protein, a MADS-box transcription factor^9^]. Biotechnology tools can complement existing selective breeding and mutagenesis techniques and allow for more efficient and targeted yield improvements in crops, which will be important for reaching global agricultural sustainability and food security goals.^10,10,11^

MADS box proteins belong to a multigene family of transcription factors found in plants, animals, and fungi^12^ and have recently been identified as candidates for targeted yield improvement.^9^ Transcription factors bind to specific DNA sequences and modulate endogenous gene expression. During the domestication of maize, MADS-box genes were likely selected through breeding because they regulate genes related to growth and development.^6,13^ For example, the SQUAMOSA (AP1-FUL) clade of MADS box genes is thought to play a role in flower and seed development^14^ and may be involved in flower development as plants transition from the vegetative to reproductive growth stages.^9,15–17^

As part of a transgenic discovery pipeline, hundreds of maize transcription factors (of which a subset were MADS box genes) were screened to identify gene targets for yield improvement. The *zmm28* gene, which belongs to the SQUAMOSA (AP1-FUL) clade of MADS box genes, is a transcription factor from maize that was selected as a candidate for targeted yield improvement.^9^ In the event of DP202216 maize, the introduced *zmm28* gene is controlled by the *Zea mays* translation initiation factor *gos2* gene promotor region (*zm-gos2;* [U.S. Patent 9115203^18^]. This promoter provides moderate constitutive expression,^9^ resulting in increased and extended expression of the introduced *zmm28* gene relative to endogenous *zmm28* gene expression. The increased and extended expression of the *zmm28* gene results in plants with enhanced grain yield potential through improved plant vigor, increased photosynthetic capacity, and enhanced nutrient utilization.^9^ DP202216 maize also expresses the maize-optimized phosphinothricin acetyltransferase gene (*mo-pat*) that was isolated from *Streptomyces viridochromogenes*. The PAT protein was used as a selectable marker during transformation and confers commercial levels of herbicide tolerance to glufosinate ammonium.

Plant vigor (e.g., increased plant height, leaf biomass, total leaf area, and early seedling vigor) is a phenotypic trait that is typically associated with plant yield. For example, early seedling vigor has been correlated with increased maize yield,^19^ and greater leaf area in developing plants can drive photosynthesis through improved interception of sunlight by the canopy.^20,21^ Likewise, since over 90% of plant biomass is accumulated through photosynthesis, an increase in plant photosynthetic rate likely promotes increased plant biomass and yield.^11^ Optimal carbon and nitrogen balance further supports plant growth and development, since increased photosynthesis drives increased demand for nitrogen.^22,23^ In DP202216 maize, plant height, leaf biomass, and total leaf area were all significantly increased, relative to controls, and there were statistically significant increases in photosynthesis-related endpoints and enzyme activities [e.g., carbon dioxide exchange rate, electron transport rate, PEPC activity, NADP-MDH activity, and PPDK activity;^9^] Nitrogen uptake, rate of nitrogen assimilation, and nitrogen reductase activity were also significantly increased in DP202216 maize plants compared with control maize plants. Collectively, these observed increases in these phenotypic, photosynthesis, and nitrogen utilization endpoints support the increased yield phenotype in the DP202216 maize plants that was observed across multiple environments, hybrid backgrounds, and years.^9^

## Hazard Assessment of the Newly Expressed Proteins

### PAT Protein

The *mo-pat* gene expresses the PAT protein, which is identical to the PAT protein expressed in number of approved GM events across several different crops that are currently in commercial use.^24^ The PAT protein has been previously assessed for potential allergenicity and toxicity and has been found unlikely to present any significant risks to the environment, human, or animal health.^25^ In addition, there is a considerable body of public information supporting the safety of the PAT protein.^25–28^ Previously, nonspecific acetylation has been reported;^29^ however, no safety implications are known. PAT protein safety has been reviewed and authorized for food and feed use by regulatory authorities in 20 different countries and/or regions. In total there are about 460 regulatory approvals in these countries, representing 7 species of plants and more than 110 transformation events.^26^ Therefore, the present review is restricted to the DP202216 maize event and the introduced ZMM28 protein.

### ZMM28 Protein

As part of the weight of evidence of safety, potential hazards of the ZMM28 protein were identified (Tier 1) by considering the source of the *zmm28* gene, the mode of action, the history of exposure to transcription factors in food, the HOSU of the ZMM28 protein, and the potential for the ZMM28 protein to be an allergen or toxin (via bioinformatics assessment of the gene sequence compared with a database of known allergens or toxins) (Table 1). The source of the *zmm28* gene is maize, which is extensively cultivated worldwide and has a long history of safe use. Maize grain and maize-derived products represent staple food and feed for a large portion of the global population.^30^ No significant toxicity has been ascribed to any food or feed uses of maize, and maize has been described as a food that is likely to have low allergenicity.^31^ The introduced *zmm28* gene is native to maize, and the mode of action of the ZMM28 protein has been characterized.^9^ The history of exposure to transcription factors in human and animal diets is well established as they are ubiquitous, endogenous proteins found in plants.^32^ The HOSU of the ZMM28 protein has also been evaluated, which supports the safety assessment of the ZMM28 protein for food and feed.^34,33^ Based on bioinformatic sequence analysis, the introduced ZMM28 amino acid sequence in DP202216 maize is identical to the native ZMM28 amino acid sequence in non-modified conventional maize, and it is also identical to the ZMM28 protein in several commonly consumed varieties of sweet corn.^33^ In addition, the ZMM28 protein shares homology with the proteins of its homologs in many other food crops.^33^

**Table 1.** Tiered weight of evidence approach used to identify (Tier I) and characterize (Tier II) potential hazards of the ZMM28 protein in DP202216 maize

	Weight of evidence	
Tier I: Hazard Identification	Source of the *zmm28* gene	The source of the *zmm28* gene is maize. The safety of maize is well established as an agricultural food and feed crop).^30,31^
	Mode of action of the ZMM28 protein	The ZMM28 protein is a MADS-box transcription factor, which are thought to play a role in plant growth and development.^6,13^ The introduced ZMM28 protein in DP202216 maize has been shown to enhance yield potential through improved plant vigor, increased photosynthetic capacity, and enhanced nutrient utilization.^9^
	History of exposure to transcription factors	There is a well-established history of exposure to transcription factors in food and feed.^32^
	History of safe use (HOSU) of the ZMM28 protein	The *zmm28* gene is native to maize. The amino acid sequence of the endogenous and the introduced ZMM28 proteins in DP202216 maize are identical. The amino acid sequence of the introduced ZMM28 protein in DP202216 maize is also identical to the amino acid sequence of the ZMM28 protein found in several varieties of sweet corn. Many other food crops, fruits and vegetables contain closely related or homologous proteins.^33^
	Potential for ZMM28 protein to be an allergen or toxin	A bioinformatics assessment of the ZMM28 protein sequence for potential cross-reactivity with known or putative allergens was conducted according to relevant guidelines.^34,35^ No alignments that were a length of 80-mers or greater with a sequence identity of ≥ 35% were identified. A bioinformatics assessment was also conducted to assess the potential toxicity of the ZMM28 protein. No alignments with an E-value ≤ 10^−4^ were returned between the ZMM28 protein sequence and any protein sequence in the internal toxin database, indicating no apparent relationship between the ZMM28 protein and known toxins. Heat stability and digestibility of the protein in simulated gastric and intestinal fluids add to the weight of evidence that a protein is unlikely to be toxic or allergenic. Because this gene is endogenous to maize, and there are no identified hazards for the ZMM28 protein, these studies are not necessary to inform an exposure assessment in support of the safety assessment.
Tier II: Hazard Characterization	Acute toxicity	When triggered by uncertainty in the Tier I assessment, additional hazard characterization studies (e.g., mouse acute oral toxicity) are typically conducted to assess the safety of newly expressed proteins. Based on the weight of evidence of safety of the ZMM28 protein, additional hazard characterization studies are not necessary to assess safety.

A bioinformatics assessment of the ZMM28 protein sequence for potential cross-reactivity with known or putative allergens was conducted according to relevant guidelines^34,35^ using the ZMM28 protein sequence [as shown in Figure 1 of^33^] and an up-to-date allergen database (Comprehensive Protein Allergen Resource (COMPARE) 2019 database (January 2019) available at http://comparedatabase.org). This peer-reviewed database is a collaborative effort of the Health and Environmental Sciences Institute (HESI) Protein Allergens, Toxins, and Bioinformatics (PATB) Committee and is comprised of 2,081 sequences. The ZMM28 protein sequence was used as the query in a FASTA v35.4.4^37,36^ search against the allergen sequences. The search was conducted using default parameters, except the *E*-score threshold was set to 10^−4^. An *E*-score threshold of 10^−4^ has been shown to be an appropriate value for allergenicity searches.^37^ No alignments that were a length of 80-mers or greater with a sequence identity of ≥35% were identified (including an adjustment for shorter alignments where ≥29 amino acid identity is present: 29/80> 35%). A second search was conducted to identify any contiguous 8-residue identical matches between the ZMM28 protein sequence and allergen sequences. No contiguous 8 residue matches were identified in the second search. Taken together, the comparison of the ZMM28 protein sequence to the allergen sequences showed that there is no apparent allergenicity concern for the ZMM28 protein. Even using these highly conservative alignment thresholds,^37,38^ no potential cross-reactive risks were identified.

A bioinformatics assessment was also used to assess the potential toxicity of the ZMM28 protein. An internal toxin database was developed based on the sequences in UniProtKB/Swiss-Prot (https://www.uniprot.org/). The internal toxin database is updated annually and includes a subset of protein sequences that are considered most relevant for assessing toxicity or adverse health effects (e.g., keywords containing toxin, hemagglutinin, vasoactive, etc.). BLASTP was used to assess alignment based on default parameters and a few exceptions (low complexity filtering was turned off, the E-value threshold was conservatively set to 10^−4^, and unlimited alignments were returned). No alignments with an E-value ≤ 10^−4^ were returned between the ZMM28 protein sequence [as shown in Figure 1 in Ref. 33] and any protein sequence in the internal toxin database, indicating no apparent relationship between the ZMM28 protein and known toxins.

Confirmation of the safety of source of the *zmm28* gene, the history of exposure to transcription factors in food, and the presence of the ZMM28 protein in sweet corn supports, in part, the evaluation of history of safe use, which can be leveraged in the safety assessment of the ZMM28 protein. Furthermore, confirmation of the lack of allergenicity or toxicity of the ZMM28 protein using *in silico* analysis adds to the weight of evidence of safety. Using this weight of evidence approach, we found that the increased and extended expression of the ZMM28 protein in DP202216 maize presents low risk for adverse health effects. In this case, additional hazard identification (e.g., heat liability, digestibility in simulated gastric fluid), and characterization studies (e.g., mouse acute oral toxicity), that are typically conducted to assess the safety of newly expressed proteins in GM crops without a history of safe use, are not necessary to assess the safety of the ZMM28 protein in DP202216 maize because the HOSU of the endogenous ZMM28 protein establishes its negligible hazard (Table 1).

## Assessment of Potential Unintended Effects on Agronomics

Typically, for cultivation, it is important to understand if the GM plant presents a risk to other plants (e.g., increased weediness, invasiveness, gene flow to sexually compatible wild relatives). Potential changes in agronomic practices (for example, increased weed resistance or changes in herbicide use) are also considered, and for a trait that confers protection against insect pests, the potential risk to non-target organisms in the environment is assessed. Problem formulation was used as a tool to formulate plausible pathways to harm, and knowledge about the basic biology of maize was used to inform the environmental risk assessment ^[39]^. Maize is highly domesticated, does not survive outside of cultivation, and is not considered an invasive plant.^30,40^ Pollen-mediated gene flow is limited in maize due to the large size of maize pollen and a limited range of dispersal.^40^ Therefore, non-GM maize is not considered weedy or invasive.

The characteristics of the introduced trait were considered to further inform the risk assessment Table 2. The ZMM28 protein is a maize transcription factor, and increased and extended expression of the introduced *zmm28* gene relative to endogenous *zmm28* gene expression results in plants with enhanced grain yield potential through improved plant vigor, increased photosynthetic capacity, and enhanced nutrient utilization.^9^ Based on the low overall expression of the introduced ZMM28 protein in DP202216 maize relative to the endogenous ZMM28 protein^9^ and the intended function of the gene, there is no plausible hypothesis to suggest that the increased and extended expression of the ZMM28 protein in DP202216 maize could result in harm to non-target organisms in the environment. On the other hand, while the ZMM28 protein is not intended to increase weediness, invasiveness, or survival characteristics of the plant, a plausible hypothesis can be developed for how increased vigor of the plant could result in increased weediness or survival characteristics Table 2.

**Table 2.** Hypothesis-driven studies to assess potential unintended effects of the ZMM28 transcription factor

**Rational for additional hypothesis-based studies**	Transcription factors modulate endogenous gene expression. Potential unintended changes in the plant due to a transcription factor can be assessed using composition, agronomic, and nutritional equivalency studies.^31^
**Composition**	There were no biologically relevant differences in the nutrient composition of forage and grain between DP202216 maize and conventional maize.^41^ These results support the conclusion that there were no unintended effects of the genetic insertion or increased expression of the endogenous ZMM28 transcription factor on the composition of the plant.
**Agronomics**	There were no biologically relevant differences in the agronomic endpoints and DP202216 maize was determined to be comparable to conventional maize (Supplement). These results support the conclusion that there were no unintended effects of the genetic insertion or increased expression of the endogenous ZMM28 transcription factor on the agronomics of plant.
**Nutritional equivalency and adverse effects**	Based on results from a 42-day broiler study and a 90-day rat study, diets produced with DP202216 maize grain were nutritionally equivalent to, and as safe as, diets produced with control maize grain.^53,54^ These results support the conclusion that maize grain containing event DP202216 is as safe and nutritious as maize grain that does not contain event DP202216.

To fulfill regulatory data requirements, an agronomic study was conducted to investigate whether the agronomics of DP212216 maize is comparable to non-GM maize. The relevant endpoints to consider related to weediness, invasiveness, and survival are early stand count, final stand count, days to flowering, and pollen viability, however, to satisfy regulatory requirements, the standard suite of agronomics endpoints was evaluated (including early stand count, days to flowering, plant height, lodging, final stand count, days to maturity, pollen viability, number of kernels, harvest grain moisture, yield, and 100-kernel weight; Supplemental Information Table S1). The multi-location field trial was conducted at 12 sites in commercial maize-growing regions of the United States and Canada (three sites in Iowa, two sites in Illinois, and one site in Indiana, Kansas, Missouri, Ontario, Nebraska, Pennsylvania, and Texas) during the 2017 growing season. The field study design and maintenance practices have been summarized previously.^41^ Test entries included one GM maize hybrid (DP202216) maize, one non-modified near-isoline control maize hybrid (control maize), and 16 non-GM commercial maize hybrids (reference maize), as described previously.^41^ Statistical analysis was conducted to compare the agronomic endpoints from DP202216 maize and control maize using SAS software, Version 9.4 (SAS Institute Inc., Cary, NC, USA). Briefly, agronomic endpoints were fit using a linear mixed model, and means were estimated for each maize line and compared to determine statistical significance (unadjusted *P*-value <0.05). The false discovery rate (FDR) method^42,43^ was used to control for false-positive outcomes across all agronomic endpoints analyzed using linear mixed models, and the adjusted *P*-values are reported for agronomic endpoints with an unadjusted *P*-value < 0.05. Statistical differences were also evaluated in the context of normal ranges of variation established from concurrently grown non-GM, commercial maize (referred to as reference maize) data, as described previously.^41^

Three agronomic endpoints (pollen shape at 60 and 120 minutes, and pollen color at 120 minutes) were not included in statistical analysis because they did not meet the minimum levels of non-uniformity (Supplemental Information Table S2). No statistical differences were identified between DP202216 maize and control maize in the across-site analysis for early stand count, days to flowering, pollen viability (based on shape at 0 and 30 minutes or color at 0, 30, and 60 minutes), plant height, days to maturity, lodging, number of kernels, harvest grain moisture, and 100-kernel weight (Supplemental Information Table S2). A statistically significant difference was detected for final stand count and yield between DP202216 maize and control maize (unadjusted *P*-value = 0.00611 and 0.00566, respectively); however, neither of these endpoints were statistically significant after FDR adjustment (FDR adjusted *P*-value = 0.0519 and 0.0519, respectively; Supplemental Information Table S2), which indicates that these differences in final stand count and yield are likely false-positive outcomes due to multiplicity. These statistically significant endpoints were further assessed for biological relevance by comparing the range of individual values from DP202216 maize to the in-study reference range. For final stand count, 47 of 48 values for DP202216 maize were within the reference data range, and one value was above the upper reference range. For yield, 46 of 48 values for DP202216 maize were within the reference data range, and two values were below the lower reference range (Supplemental Information Table S2). The results obtained in the field study demonstrated that agronomic characteristics of DP202216 maize were comparable to those of conventional maize, and the minor differences observed for final stand count and yield are unlikely to result in DP202216 maize plants with increased weediness potential or survivability, compared to conventional maize. Therefore, the environmental risks associated with the cultivation of DP202216 maize are low. While yield in this regulatory field study was comparable to control maize, enhanced yield potential for DP202216 maize was observed in field evaluations containing a larger number genotypes and a significantly larger number of replications across multiple years and sites.^9^

## Assessment of Potential Unintended Effects on Composition

A compositional assessment is part of the weight-of-evidence approach used to evaluate the food and feed safety of genetically modified plant products.^44,45^ Compositional assessment is currently required for regulatory approval by many global authorities; however, comprehensive literature is available to support the conclusion that genetically modified plants are no more likely to have unintended changes in composition than traditionally bred plants.^46,47^ Therefore, composition analysis should only be conducted when a plausible hypothesis can be developed for changes in composition based on the trait, mode of action, etc.^48^ In the case of DP202216 maize, the expression of the endogenous ZMM28 transcription factor was increased and extended, and because transcription factors modulate endogenous gene expression, we investigated if there were unintended changes in the composition of DP202216 maize grain or forage Table 2. As summarized by,^41^ there were no biologically relevant differences in the nutrient composition of forage and grain derived between DP202216 maize and conventional maize, which adds to the weight of evidence for the safety of DP202216 maize.

## Assessment of Potential Unintended Effects on Nutrition Using Whole-food Animal Feeding Studies

Animal feeding studies are required by some regulatory authorities to investigate if there are unintended changes in the nutrition of the GM grain^49,50^ or potential adverse effects. However, the scientific rationale for requiring these studies and the ethics of conducting animal feeding studies to assess nutrition of grain has been challenged based on the large number of studies that have concluded that GM crops do not have unexpected changes in nutrition that would result in harm.^49,51,52^ In the case of DP202216 maize, based on the lack of observed differences in the composition of grain or forage, the likelihood of observing adverse effects in DP202216 maize grain nutrition was low. However, to satisfy global data requirements, the required animal feeding studies were conducted to investigate the nutritional equivalence and safety of DP202216 maize grain Table 2.

A 42-day broiler study was conducted with male and female Ross 708 broiler chickens.^53^ No statistical differences (*P*-value > 0.05) were observed between broilers consuming diets produced with DP202216 test maize grain and broilers consuming diets produced with control maize grain for 42 days (for standard measurement endpoints including weight gain, mortality, mortality-adjusted feed efficiency, organ yields, or carcass parts yields).^53^ Based upon the results from this study, diets produced with DP202216 maize grain were determined to be nutritionally equivalent to diets produced with control maize grain.

A 90-day rat feeding study was conducted to satisfy regulatory requirements (for example, Implementing Regulation (EU) No 503/2013). No statistical differences (*P*-value>0.05) were observed between rats consuming diets produced with over 50% DP202216 maize grain and rats consuming diets produced with control maize grain for 90 days (for standard measurement endpoints including body weight, body weight gain, food consumption, food efficiency, clinical signs of toxicity, neurobehavioral assessments, clinical pathology parameters, organ weights, or gross or microscopic pathology).^54^ These results support the conclusion that maize grain containing event DP202216 is as safe and nutritious as maize grain that does not contain event DP202216.

## Conclusions

Over the past 25 years, there have been over 4000 GM events assessed for safety in 70 countries, and the safety assessment framework is well-established. However, the studies required to support safety assessments are often driven by regulatory requirements dictated by global authorities in response to policies designed to appease perceived public fear, rather than a scientific, hypothesis-based approach.^55^ Use of a science-based approach, which considers the weight of evidence (WOE) for overall conclusions regarding safety, is more efficient and effective for assessing risk because only those studies that directly inform the risk assessment are conducted. In many cases, not all Tier I and Tier II data are scientifically justified to assess the safety of a newly expressed protein. Often, some data routinely required by regulatory authorities is unnecessary to assess GM crop safety (for example, dietary exposure assessments, compositional analyses of forage and grain, 42-day poultry and 90-day rat feeding studies to assess nutritional equivalence of grain or potential for adverse effects, and mouse 14-day acute oral or 28-day repeated dose studies to assess toxicity of the newly introduced protein). As originally proposed by Delaney et al., these studies should only be required on a case-by-case basis when these data are informative to the overall risk assessment.^4^

A science-based, WOE approach was used to assess the food, feed, and environmental safety of DP202216 maize, and this review of the safety of DP202216 maize is summarized to serve as a reference for regulatory agencies and decision-makers in countries where authorization of DP202216 maize will be pursued, and for others interested in food, feed, and environmental safety. As part of this assessment, problem formulation was used to identify potential pathways to harm and inform the design and conduct of studies that were relevant to the risk assessment. In the case of DP202216 maize, it was concluded that not all Tier I and Tier II data were necessary to assess hazard of the ZMM28 protein. For example, the ZMM28 protein in DP202216 maize is identical to the endogenous ZMM28 protein present in conventional maize and sweet corn.^33^ And, based on the safety of the source of the *zmm28* gene, HOSU of the ZMM28 protein, and lack of allergenicity or toxicity observed using *in silico* bioinformatics analysis of the deduced amino acid sequence, the ZMM28 protein is unlikely to be either allergenic or toxic to humans. Additional assessment of heat stability, digestion, or acute toxicity of the ZMM28 protein was not necessary for hazard identification and characterization. While poultry and rat grain feeding studies were conducted to comply with regulatory requirements, these studies were considered supplementary based on the lack of differences observed in the compositional assessment of grain and forage. As expected, animal feeding studies using broiler chickens^53^ and rats^54^ demonstrated low likelihood of unintended effects on nutrition or safety. Publishing the results of these studies is intended to add to the scientific literature demonstrating the safety and lack of unexpected differences in nutrition between GM and non-modified crop grain, and further supports the position that these studies should be required on a case-by-case basis when needed to inform the risk assessment.^4^ Collectively, the weight of evidence for event DP202216 maize indicates low potential for adverse effects on humans, animals, or the environment.

## Supplementary Material

Supplemental Material

## Supplemental Information Description

Tables summarizing the agronomic characteristics of DP202216 maize.

## References

[1] Codex Alimentarius Commission. Foods derived from modern biotechnology. 2nd ed. Food and Agriculture Organization of the United Nations, Rome, Italy: World Health Organization; 2009.

[2] EFSA. Guidance document of the scientific panel on generically modified organisms for the risk assessment of genetically modified plants and derived food and feed. Efsa J 2006;99:1–100.

[3] FAO/WHO. 1991. Strategies for assessing the safety of foods produced by biotechnology. Report of Joint FAO/WHO Consultation. Geneva, Switzerland

[4] Delaney B, Amanda Shen Z, Powley CR, Gannon S, Munley SA, Maxwell C, Barnett JF Jr. Acute and repeated dose oral toxicity of *N*-acetyl-L-aspartic acid in Sprague-Dawley rats. Food Chem Toxicol. 2008;46:2023–34. doi:10.1016/j.fct.2008.01.042. 18329151

[5] Duvick DN. The contribution of breeding to yield advances in maize (*Zea mays* L.). Adv Agron. 2005;86:83–145.

[6] Schilling S, Pan S, Kennedy A, Melzer R. MADS-box genes and crop domestication: the jack of all traits. J Exp Bot. 2018;69:1447–69. 29474735 10.1093/jxb/erx479

[7] Oladosu Y, Rafii MY, Abdullah N, Hussin G, Ramli A, Rahim HA, Miah G, Usman M. Principle and application of plant mutagenesis in crop improvement: a review. Biotechnol Biotechnol Equip. 2016;30:1–16. doi:10.1080/13102818.2015.1087333.

[8] Qi Y. The breeding of maize inbred line Fu746 by radiation induced mutations and its application. Acta Agriculturae Nucleatae Sinica. 2006;20:174–76.

[9] Wu J, Lawit SJ, Weers B, Sun J, Mongar N, Van Hemert J, Melo R, Meng X, Rupe M, Clapp J, et al. Overexpression of *zmm28* increases maize grain yield in the field. Proc National Acad Sci USA. 2019;116: 23850–58 10.1073/pnas.1902593116 PMC6876154 31685622

[10] Makino A. Photosynthesis, grain yield, and nitrogen utilization in rice and wheat. Plant Physiol. 2011;155:125–29. doi:10.1104/pp.110.165076. 20959423 PMC3014227

[11] Ort DR, Merchant SS, Alric J, Barkan A, Blankenship RE, Bock R, Croce R, Hanson MR, Hibberd JM, Long SP, et al. (2015) Redesigning photosynthesis to sustainably meet global food and bioenergy demand. Proceedings of the National Academy of Sciences 112: 8529–36 10.1073/pnas.1424031112 PMC4507207 26124102

[12] Becker A, Theißen G. The major clades of MADS-box genes and their role in the development and evolution of flowering plants. Mol Phylogenet Evol. 2003;29:464–89. doi:10.1016/S1055-7903(03)00207-0. 14615187

[13] Zhao Q, Weber AL, McMullen MD, Guill K, Doebley J. MADS-box genes of maize: frequent targets of selection during domestication. Genet Res (Camb). 2011;93:65–75. doi:10.1017/S0016672310000509. 21144126 PMC3474543

[14] Theissen G, Becker A, Di Rosa A, Kanno A, Kim JT, Münster T, Winter K-U, Saedler H. A short history of MADS-box genes in plants. Plant Mol Biol. 2000;42:115–49. doi:10.1023/A:1006332105728. 10688133

[15] Ferrandiz C, Gu Q, Martienssen R, Yanofsky MF. Redundant regulation of meristem identity and plant architecture by FRUITFULL, APETALA1 and CAULIFLOWER. Development. 2000;127:725–34. 10648231 10.1242/dev.127.4.725

[16] Hartmann U, Höhmann S, Nettesheim K, Wisman E, Saedler H, Huijser P. Molecular cloning of *SVP*: a negative regulator of the floral transition in *Arabidopsis*. Plant J. 2000;21:351–60. doi:10.1046/j.1365-313x.2000.00682.x. 10758486

[17] Purugganan MD, Rounsley SD, Schmidt RJ, Yanofsky MF. Molecular evolution of flower development: diversification of the plant MADS-box regulatory gene family. Genetics. 1995;140:345–56. 7635298 10.1093/genetics/140.1.345 PMC1206560

[18] Taramino G, Sakai H, Tingey SV, Luck S, Tomes D, Niu X, inventors. Plants with altered root architecture, related constructs and methods involving genes encoding exostosin family polypeptides and homologs thereof. United States Patent, Patent No. US 9,115,203 B2; Aug 25 2015.

[19] Trachsel S, Burgueno J, Suarez EA, San Vicente FM, Rodriguez CS, Dhliwayo T. Interrelations among early vigor, flowering time, physiological maturity, and grain yield in tropical maize (*Zea mays* L.) under multiple abiotic stresses. Crop Sci. 2017;57:229–42. doi:10.2135/cropsci2016.06.0562.

[20] Duncan WG. Leaf angles, leaf area, and canopy photosynthesis. Crop Sci. 1971;11:482–85. doi:10.2135/cropsci1971.0011183X001100040006x.

[21] Stewart DW, Costa C, Dwyer LM, Smith DL, Hamilton RI, Ma BL. Canopy Structure, Light Interception, and Photosynthesis in Maize. Agron J. 2003;95:1465–74. doi:10.2134/agronj2003.1465.

[22] Paul MJ, Pellny TK. Carbon metabolite feedback regulation of leaf photosynthesis and development. J Exp Bot. 2003;54:539–47. doi:10.1093/jxb/erg052. 12508065

[23] Zheng Z-L. Carbon and nitrogen nutrient balance signaling in plants. Plant Signal Behav. 2009;4:584–91. doi:10.4161/psb.4.7.8540. 19820356 PMC2710548

[24] ISAAA. GM approval database. International Service for the Acquisition of Agri-Biotech Applications. Ithaca, New York, USA; 2019. https://www.isaaa.org/gmapprovaldatabase/default.asp

[25] OECD. 1999. Consensus document on general information concerning the genes and their enzymes that confer tolerance to phosphinothricin herbicide. Organisation for Economic Co-operation and Development, ENV/JM/MONO(99)13, Paris, France.

[26] CERA - ILSI Research Foundation. A review of the food and feed safety of the PAT protein. International Life Sciences Institute, Center for Environmental Risk Assessment; 2016. http://ilsirf.org/publication/a-review-of-the-food-and-feed-safety-of-the-pat-protein/

[27] CERA. A review of the environmental safety of the PAT protein. Environ Biosafety Res Oct-Dec 2011;10(4):73–101. doi:10.1051/ebr/2012004 22781085

[28] Hérouet C, Esdaile DJ, Mallyon BA, Debruyne E, Schulz A, Currier T, Hendrickx K, van der Klis R-J, Rouan D. Safety evaluation of the phosphinothricin acetyltransferase proteins encoded by the *pat* and *bar* sequences that confer tolerance to glufosinate-ammonium herbicide in transgenic plants. Regul Toxicol Pharmacol. 2005;41:134–49. doi:10.1016/j.yrtph.2004.11.002. 15698537

[29] Christ B, Hochstrasser R, Guyer L, Francisco R, Aubry S, Hörtensteiner S, Weng J-K. Non-specific activities of the major herbicide-resistance gene BAR. Nat Plants. 2017;3.12:937–45. doi:10.1038/s41477-017-0061-1. 29180815 PMC6342461

[30] CFIA. 1994. The biology of *Zea mays* (L.) (Maize). Canadian Food Inspection Agency, BIO1994-11.

[31] OECD. 2002. Consensus document on compositional considerations for new varieties of maize (*Zea Mays*): key food and feed nutrients, anti-nutrients and secondary plant metabolites. Organisation for Economic Co-operation and Development, ENV/JM/MONO(2002)25.

[32] Parrott W, Chassy B, Ligon J, Meyer L, Petrick J, Zhou J, Herman R, Delaney B, Levine M. Application of food and feed safety assessment principles to evaluate transgenic approaches to gene modulation in crops. Food Chem Toxicol. 2010;48:1773–90. doi:10.1016/j.fct.2010.04.017. 20399824

[33] Anderson J, Brustkern S, Cong B, Deege L, Delaney B, Hong B, Lawit S, Mathesius C, Schmidt J, Wu J, et al. Evaluation of the history of safe use of the maize ZMM28 protein. J Agric Food Chem. 2019a;67:7466–74. doi:10.1021/acs.jafc.9b00391. 31184886

[34] Codex Alimentarius Commission. Alinorm 03/34: appendix III: draft guideline for the conduct of food safety assessment of foods derived from recombinant-DNA plants, and Appendix IV: proposed draft annex of the assessment of possible allergenicity. Rome: Food and Agriculture Organization of the United Nations, World Health Organization; Rome, Italy: 2003. p. 47–60.

[35] FAO/WHO. Evaluation of allergenicity of genetically modified foods: report of a joint FAO/WHO expert consultation on allergenicity of foods derived from biotechnology, 22-25 January 2001. Rome: Food and Agriculture Organization of the United Nations; Rome, Italy: 2001.

[36] Pearson WR, Lipman DJ Improved tools for biological sequence comparison. Proc National Acad Sci. 1988;85: 2444–48 10.1073/pnas.85.8.2444 PMC280013 3162770

[37] Mirsky HP, Cressman RF Jr, Ladics GS. Comparative assessment of multiple criteria for the in silico prediction of cross-reactivity of proteins to known allergens. Regul Toxicol Pharmacol. 2013;67:232–39. doi:10.1016/j.yrtph.2013.08.001. 23933007

[38] Silvanovich A, Bannon G, McClain S. The use of *E*-scores to determine the quality of protein alignments. Regul Toxicol Pharmacol. 2009;54:S26–S31. doi:10.1016/j.yrtph.2009.02.004. 19245824

[39] Anderson J, Bachman P, Burns A, Chakravarthy S, Goodwin L, Privalle L, Song S, Storer N. Streamlining data requirements for the environmental risk assessment of genetically modified (GM) crops for cultivation approvals. J Regulat Sci. 2020;8:1–12 (IN PRESS).

[40] OECD. 2003. Consensus document on the biology of *Zea mays* subsp. *mays* (Maize). Organisation for Economic Co-operation and Development, ENV/JM/MONO(2003)11.

[41] Anderson JA, Hong B, Moellring E, TeRonde S, Walker C, Wang Y, Maxwell C. Composition of forage and grain from genetically modified DP202216 maize is equivalent to non-modified conventional maize (*Zea mays* L.). GM Crops Food. 2019b;10:77–89. doi:10.1080/21645698.2019.1609849. 31094289 PMC6615539

[42] Benjamini Y, Hochberg Y. Controlling the false discovery rate: a practical and powerful approach to multiple testing. J R Stat Soc B. 1995;57:289–300.

[43] Westfall PH, Tobias RD, Rom D, Wolfinger RD, Hochberg Y. Concepts and basic methods for multiple comparisons and tests. in multiple comparisons and multiple tests: using SAS. Cary: SAS Institute Inc.; 1999. 13–40.

[44] Codex Alimentarius Commission. 2008. Guideline for the conduct of food safety assessment of foods derived from recombinant-DNA plants. Codex Alimentarius, CAC/GL 45-2003. Rome, Italy.

[45] OECD. 1993. Safety evaluation of foods derived by modern biotechnology: concepts and principles. Organisation for Economic Cooperation and Development. Paris, France.

[46] Herman RA, Price WD. Unintended compositional changes in genetically modified (GM) crops: 20 years of research. J Agric Food Chem. 2013;61:11695–701. doi:10.1021/jf400135r. 23414177

[47] Schnell J, Steele M, Bean J, Neuspiel M, Girard C, Dormann N, Pearson C, Savoie A, Bourbonnière L, Macdonald P. A comparative analysis of insertional effects in genetically engineered plants: considerations for pre-market assessments. Transgenic Res. 2015;24:1–17. doi:10.1007/s11248-014-9843-7. 25344849 PMC4274372

[48] Herman RA, Chassy BM, Parrott W. Compositional assessment of transgenic crops: an idea whose time has passed. Trends Biotechnol. 2009;27:555–57. doi:10.1016/j.tibtech.2009.07.003. 19699543

[49] Bartholomaeus A, Parrott W, Bondy G, Walker K. The use of whole food animal studies in the safety assessment of genetically modified crops: limitations and recommendations. Crit Rev Toxicol. 2013;43:1–24. doi:10.3109/10408444.2013.842955. PMC3833814 24164514

[50] Mendelsohn M, Kough J, Vaituzis Z, Matthews K. Are *Bt* crops safe? Nat Biotechnol. 2003;21:1003–09. doi:10.1038/nbt0903-1003. 12949561

[51] Devos Y, Gaugitsch H, Gray AJ, Maltby L, Martin J, Pettis JS, Romeis J, Rortais A, Schoonjans R, Smith J, et al. Advancing environmental risk assessment of regulated products under EFSA’s remit. Efsa J. 2016;14:e00508. doi:10.2903/j.efsa.2016.s0508.

[52] Herman RA, Ekmay R. Do whole-food animal feeding studies have any value in the safety assessment of GM crops? Regul Toxicol Pharmacol. 2014;68:171–74. doi:10.1016/j.yrtph.2013.07.003. 23851038

[53] McNaughton J, Roberts M, Smith M, Carlson A, Mathesius C, Roper J, Zimmermann C, Walker C, Huang E, Herman R. Evaluation of broiler performance and carcass yields when fed diets containing maize grain from transgenic product DP-2Ø2216-6. J Appl Poult Res. 2020;29:700–11. doi:10.1016/j.japr.2020.05.004.

[54] Carlson A, Pushkor Mukerji B, Mathesius CA, Huang E, Herman RA, Hoban D, Thurman JD, Roper JM. DP-2Ø2216-6 maize does not adversely affect rats in a 90-day feeding study. Regul Toxicol Pharmacol: 104779. 2020;117:104779. doi:10.1016/j.yrtph.2020.104779. 32888975

[55] Herman RA, Gaffney J, Storer NP. Enlightened oversight of genetically engineered crops for the next generation. Agric Environ Lett. 2020;5:e20004. doi:10.1002/ael2.20004.

