# Supplementary material for: Hypothesis-based food, feed, and environmental safety assessment of GM crops: A case study using maize event DP-202216-6
Source: GM Crops Food. 2021 Jan 21;12(1):282–91. doi: 10.1080/21645698.2020.1869492 (PMC7833765; doi:10.1080/21645698.2020.1869492)
Supplement: Supplemental Material [file KGMC_A_1869492_SM0557.docx]

**Hypothesis-based Food, Feed, and Environmental Safety Assessment of GM Crops: A Case Study Using Maize Event DP-2Ø2216-6**

Jennifer A. Anderson, Rod Herman, Anne Carlson, Carey Mathesius, Carl Maxwell, Henry Mirsky, Jason Roper, Brenda Smith; Carl Walker; Jingrui Wu

Corteva Agriscience™, 7300 NW 62nd Avenue, Johnston, IA 50131, USA

*Corresponding Author

Jennifer A. Anderson

Corteva Agriscience™

Johnston, IA 50131, USA

[jennifer.anderson@corteva.com](mailto:jennifer.anderson@corteva.com)

(515) 535-3730 (phone)

###### Supplement Table 1. Description of Agronomic Characteristics Evaluated

| **Characteristic Measured** | **Evaluation Timing^a^** | **Description** |
| --- | --- | --- |
| Early Stand Count | V2-V4 | Number of plants emerged per plot (count/m^2^) |
| Days to Flowering | Approximately 50% pollen shed | From the time of planting until approximately 50% of the plants have tassels shedding pollen (days) |
| Plant Height | R4 | Height from soil surface to the collar of flag leaf (cm) |
| Lodging | R6 | Combined score of stalk lodging (the number of plants in each plot with stalks broken below the primary ear) and root lodging (the number of plants in each plot with stalks leaning approximately 45 degrees or more) values (percentage) |
| Final Stand Count | R6 | Number of plants remaining per plot (count) |
| Days to Maturity | Physiological maturity | Number of days for the majority of the plants to first reach physiological maturity (days) |
| Pollen Viability^b^ | During pollen shed | Pollen Shape: pollen grains with collapsed walls at 0, 30, 60, and 120 minutes (% of pollen with collapsed walls) |
|  |  | Pollen Color: pollen grains with intense yellow color at 0, 30, 60, and 120 minutes (% of pollen yellow in color) |
| Number of Kernel Rows per Ear | Post harvest | Total number of kernel rows per ear (average from five primary ears) |
| Number of Kernels per Row | Post harvest | Total number of kernels per row (five primary ears for four rows) |
| Number of Kernels per Ear | Post harvest | Kernel rows per ear multiplied by average number of kernels per row (five primary ears for four rows) |
| Harvest Grain Moisture | Approximately R6 | Grain moisture content (percent) |
| Yield | Approximately R6 | Harvest weight per area adjusted to 15.5% moisture content (bushels per acre) |
| 100-Kernel Weight | Post harvest | Weight of 100 kernels (grams) |

^a^ Refer to [Abendroth *et al.* (2011)](#_ENREF_1) for a description of maize growth stages.

^b^ Pollen viability has been correlated to pollen shape and color ([Luna *et al.*, 2001](#_ENREF_43)).

###### Supplement Table 2. Across-Site Analysis of Agronomic Characteristics Results

| **Agronomic Characteristic** | **Reported Statistics** | **Control Maize** | **DP202216 Maize** | **Reference Data Range** |
| --- | --- | --- | --- | --- |
| Early Stand (count/m^2^) | Mean | 6.2 | 6.2 | 4.6 - 6.6 |
|  | Range | 5.7 - 6.9 | 5.1 - 6.8 |  |
|  | Confidence Interval | 6.1 - 6.4 | 6.0 - 6.3 |  |
|  | Adjusted P-Value | -- | 0.272 |  |
|  | P-Value | -- | 0.0534 |  |
| Days to Flowering (days) | Mean | 62.6 | 62.7 | 53 - 74 |
|  | Range | 54 - 72 | 55 - 74 |  |
|  | Confidence Interval | 59.5 - 65.8 | 59.6 - 65.9 |  |
|  | Adjusted P-Value | -- | 0.803 |  |
|  | P-Value | -- | 0.601 |  |
| Pollen Viability-Shape, 0 minutes  (% of pollen with collapsed walls) | Mean | 7.4 | 6.4 | 0 - 50 |
|  | Range | 0 - 60 | 0 - 50 |  |
|  | Confidence Interval | 1.9 - 16.1 | 1.4 - 14.7 |  |
|  | Adjusted P-Value | -- | 0.640 |  |
|  | P-Value | -- | 0.377 |  |
| Pollen Viability-Shape, 30 minutes  (% of pollen with collapsed walls) | Mean | 58.5 | 58.7 | 5 - 100 |
|  | Range | 5 - 100 | 10 - 100 |  |
|  | Confidence Interval | 38.9 - 78.2 | 39.0 - 78.4 |  |
|  | Adjusted P-Value | -- | 0.921 |  |
|  | P-Value | -- | 0.921 |  |
| Pollen Viability-Shape, 60 minutes  (% of pollen with collapsed walls) | Mean | 86.5 | 89.1 | 20 - 100 |
|  | Range | 20 - 100 | 25 - 100 |  |
|  | Confidence Interval | NA | NA |  |
|  | Adjusted P-Value | -- | NA |  |
|  | P-Value | -- | NA |  |
| Pollen Viability-Shape, 120 minutes  (% of pollen with collapsed walls) | Mean | 96.6 | 96.9 | 60 - 100 |
|  | Range | 65 - 100 | 60 - 100 |  |
|  | Confidence Interval | NA | NA |  |
|  | Adjusted P-Value | -- | NA |  |
|  | P-Value | -- | NA |  |
| Pollen Viability-Color, 0 minutes  (% of pollen yellow in color) | Mean | 8.7 | 7.4 | 0 - 60 |
|  | Range | 0 - 60 | 0 - 60 |  |
|  | Confidence Interval | 2.6 - 17.9 | 1.9 - 16.0 |  |
|  | Adjusted P-Value | -- | 0.640 |  |
|  | P-Value | -- | 0.355 |  |
| Pollen Viability-Color, 30 minutes  (% of pollen yellow in color) | Mean | 58.6 | 58.1 | 5 - 100 |
|  | Range | 5 - 100 | 5 - 100 |  |
|  | Confidence Interval | 39.7 - 77.6 | 39.1 - 77.1 |  |
|  | Adjusted P-Value | -- | 0.888 |  |
|  | P-Value | -- | 0.783 |  |
| Pollen Viability-Color, 60 minutes  (% of pollen yellow in color) | Mean | 83.4 | 86.8 | 10 - 100 |
|  | Range | 20 - 100 | 20 - 100 |  |
|  | Confidence Interval | 70.6 - 96.3 | 73.9 - 99.6 |  |
|  | Adjusted P-Value | -- | 0.552 |  |
|  | P-Value | -- | 0.195 |  |
| Pollen Viability-Color, 120 minutes  (% of pollen yellow in color) | Mean | 95.0 | 94.9 | 40 - 100 |
|  | Range | 45 - 100 | 40 - 100 |  |
|  | Confidence Interval | NA | NA |  |
|  | Adjusted P-Value | -- | NA |  |
|  | P-Value | -- | NA |  |

**Supplement Table 2. Across-Site Analysis of Agronomic Characteristics Results (continued)**

| **Agronomic Characteristic** | **Reported Statistics** | **Control Maize** | | | **DP202216 Maize** | **Reference Data Range** |
| --- | --- | --- | --- | --- | --- | --- |
| Plant Height (cm) | Mean | 224.5 | | | 223.0 | 170.0 - 311.8 |
|  | Range | 171.0 - 279.4 | | | 169.2 - 287.6 |  |
|  | Confidence Interval | 208.0 - 240.9 | | | 206.6 - 239.5 |  |
|  | Adjusted P-Value | -- | | | 0.640 |  |
|  | P-Value | -- | | | 0.294 |  |
| Days to Maturity (days) | Mean | 130.8 | | | 129.6 | 114 - 164 |
|  | Range | 111 - 164 | | | 111 - 168 |  |
|  | Confidence Interval | 122.6 - 139.0 | | | 121.3 - 137.8 |  |
|  | Adjusted P-Value | -- | | | 0.272 |  |
|  | P-Value | -- | | | 0.0686 |  |
| Lodging (%) | Mean | 1.1 | | | 1.5 | 0.0 - 16.2 |
|  | Range | 0.0 - 8.5 | | | 0.0 - 12.4 |  |
|  | Confidence Interval | NA | | | NA |  |
|  | Adjusted P-Value | -- | | | 0.272 |  |
|  | P-Value | -- | | | 0.0799 |  |
| Final Stand Count (count/m^2^) | Mean | 6.2 | | | 6.0 | 4.8 - 6.5 |
|  | Range | 5.7 - 6.5 | | | 5.1 - 6.6 |  |
|  | Confidence Interval | 6.0 - 6.3 | | | 5.9 - 6.2 |  |
|  | Adjusted P-Value | -- | | | 0.0519 |  |
|  | P-Value | -- | | | 0.00611^*^ |  |
| Number of Kernel Rows per Ear | Mean | 16.8 | | | 16.7 | 12 - 20 |
|  | Range | 14 - 18 | | | 15 - 18 |  |
|  | Confidence Interval | 16.4 - 17.2 | | | 16.3 - 17.1 |  |
|  | Adjusted P-Value | -- | | | 0.803 |  |
|  | P-Value | -- | | | 0.614 |  |
| Average Number of Kernels per Row | Mean | 37.3 | | | 37.5 | 29 - 47 |
|  | Range | 28 - 43 | | | 31 - 43 |  |
|  | Confidence Interval | 35.4 - 39.1 | | | 35.6 - 39.3 |  |
|  | Adjusted P-Value | -- | | | 0.874 |  |
|  | P-Value | -- | | | 0.720 |  |
| Number of Kernels per Ear | Mean | 625.1 | | | 626.0 | 435 - 782 |
|  | Range | 464 - 731 | | | 488 - 752 |  |
|  | Confidence Interval | 587.3 - 662.9 | | | 588.2 - 663.7 |  |
|  | Adjusted P-Value | -- | | | 0.921 |  |
|  | P-Value | -- | | | 0.903 |  |
| Harvest Grain Moisture (%) | Mean | 17.2 | | | 17.2 | 10.5 - 27.2 |
|  | Range | 10.2 - 23.3 | | | 10.2 - 23.7 |  |
|  | Confidence Interval | 15.2 - 19.3 | | | 15.1 - 19.3 |  |
|  | Adjusted P-Value | -- | | | 0.803 |  |
|  | P-Value | -- | | | 0.596 |  |
| Yield (bu/A) | Mean | 201.7 | | | 193.8 | 102 - 292 |
|  | Range | 117 - 272 | | | 85 - 261 |  |
|  | Confidence Interval | 176.9 - 226.5 | | | 169.0 - 218.5 |  |
|  | Adjusted P-Value | -- | | | 0.0519 |  |
|  | P-Value | -- | | | 0.00566^*^ |  |
| 100-Kernel Weight (g) | Mean | 36.3 | | | 35.8 | 22.9 - 45.9 |
|  | Range | 25.7 - 41.9 | | | 25.9 - 43.4 |  |
|  | Confidence Interval | 34.0 - 38.6 | | | 33.5 - 38.1 |  |
|  | Adjusted P-Value | -- | | | 0.640 |  |
|  | P-Value | -- | | | 0.346 |  |
| Note: Not applicable (NA); mixed model analysis was not performed. | | | | |  |  |
| ^*^ A statistically significant difference (P-value < 0.05) was observed. | | | |  |  |  |
